# The sensitivity of ArcCHECK‐based gamma analysis to manufactured errors in helical tomotherapy radiation delivery

**DOI:** 10.1120/jacmp.v16i1.4814

**Published:** 2015-01-08

**Authors:** Alistair K. Templeton, James C. H. Chu, Julius V. Turian

**Affiliations:** ^1^ Department of Radiation Oncology Rush University Medical Center Chicago IL USA

**Keywords:** ArcCHECK, tomotherapy, DQA, gamma analysis

## Abstract

Three‐dimensional measurement arrays are an efficient means of acquiring a distribution of data for patient plan delivery QA. However, the tie between plan integrity and traditional gamma‐based analysis of these data are not clear. This study explores the sensitivity of such analysis by creating errors in Helical Tomotherapy delivery and measuring the passing rates with an ArcCHECK cylindrical diode array. Errors were introduced in each of the couch speed, leaf open time, and gantry starting position in increasing magnitude while the resulting gamma passing rates were tabulated. The error size required to degrade the gamma passing rate to 90% or below was on average a 3% change in couch speed, 5° in gantry synchronization, or a 5 ms in leaf closing speed for a 3%/3 mm Van Dyk gamma analysis. This varied with plan type, with prostate plans exhibiting less sensitivity than head and neck plans and with gamma analysis criteria, but in all cases the error magnitudes were large compared to actual machine tolerances. These findings suggest that the sensitivity of ArcCHECK‐based gamma analysis to single‐mode errors in tomotherapy plans is dependent upon plan and analysis type and at traditional passing thresholds unable to detect small defects in the plan.

PACS number: 87.55.Qr

## I. INTRODUCTION

As complex treatment planning and delivery modalities are adopted as large components of external‐beam radiation therapy programs, patient‐specific delivery quality assurance (DQA) is necessarily also increasing in frequency and complexity. Inversely optimized treatment plans often generate beam apertures which are highly complex and bear little resemblance to commissioned fields. Thus DQA is used to verify both the dose calculation accuracy of the treatment planning system and the delivery accuracy of the treatment machine. Three‐dimensional diode/chamber arrays are attractive as they can sample a large number and spatial distribution of dose points with easy setup and data acquisition, but their role in the DQA process has not been as extensively characterized as classically used methods, specifically in conjunction with helical TomoTherapy (Accuray, Sunnyvale, CA) delivery. In this study, we explored the use of the popular VMAT QA device ArcCHECK (Sun Nuclear, Melbourne, FL) with tomotherapy, and attempted to elucidate the effects of delivery errors on DQA results.

The traditional approach to patient‐specific tomotherapy DQA is to sandwich a piece of radiographic or radiochromic film between the two halves of a homogenous, cylindrical “cheese” phantom for measurements of one plane of dose, and use one or more ion chambers inserted into holes in the phantom to sample off‐plane points.^(1)^ The plan is transposed within the treatment planning system onto the phantom, and the same dose points are calculated. The results are typically analyzed with a “gamma” type analysis,^(2)^ whereby each measured dose point is compared to the calculated dose, looking for a similar dose within a search distance of fixed size (3%/3 mm being a typical limit). Passing rates of such analyses are well‐documented.^(3)^ A similar concept is typically employed with ArcCHECK, but rather than a flat plane through the center of the phantom, many dose points are sampled in a spiral equidistant from the axis of the cylindrical phantom. While the principles are the same and the process has previously been validated for DQA measurements,^(4)^ the connection between plan versus delivery accuracy and the gamma type analysis is less intuitive, and it is unclear what types and magnitude of errors would be reflected in a drop in gamma passing rate.

Previous analyses of this combination of linear accelerator and DQA devices have tabulated passing rates on clinically approved treatment plans, which is invaluable in establishing standard deviation‐based passing criteria.^3^, ^5^, ^6^ The most straightforward sensitivity test is to alter the position of the QA device relative to the isocenter.^7^, ^8^ However, this error is one that we hope to compensate for, both with planning treatment volume (PTV) margins and image guided positioning. Garcia‐Vincente et al.,^(9)^ on the other hand, introduced random errors in the gantry angle and MLC positions of a gantry‐based static IMRT delivery to see whether they would be detected. In the present study, errors were introduced into the actual treatment delivery through the couch speed, leaf open versus closed time, and gantry synchronization to test the sensitivity of ArcCHECK gamma analysis in detection of these errors. The question we hope to address is: What magnitude do perturbations to the plan need to be before they can be reliably detected by our clinical implementation of this particular device?

## II. Materials and Methods

Nine clinically delivered patient plans were used for this study: head and neck (3), prostate with pelvic nodes (3), and prostate‐only (3), planned by various dosimetry staff. All plans were planned using the TomoTherapy treatment planning system (version 4.0.4). The ArcCHECK QA device used for measurements in this study is a cylindrical structure with 1386 diodes arranged in a helix with a diameter and length of 21 cm. The device was configured with a solid PMMA insert (no central ion chamber) and scanned with 2 mm slices on a Phillips Brilliance 16‐slice computed tomography (CT) scanner (Phillips, Amsterdam, Netherlands). For each plan, the patient image was replaced with the image of the ArcCHECK device to create a DQA plan. The device was placed in the plan space such that the diodes passed through the high‐dose region of the plan, which results in an off‐centered location of the phantom. The dose grid on the heterogeneous phantom images from the CT was then calculated using the clinical plan beam data and a “fine” dose grid ($2.34\times 2.34\times 2\;{\text{mm}}^{3}$ voxels across a 60 cm field of view), and transferred to the SNC patient software (version 6.1.0; Sun Nuclear, Melbourne, FL) which then un‐wraps the 3D dose grid into a 2D map of expected diode readings. This map is used as the reference dose for all gamma comparisons for that patient.

Each delivery plan is defined by the following parameters: field width, gantry period, gantry starting position, couch speed, and a sinogram which denotes the leaf openings over time. During delivery, these are all tied together by time, so the simulated errors are in the synchronization of these parameters. Specifically, the parameters altered were couch speed, gantry start position, and leaf open time. All but the last are simple changes in planned values, but leaf open time requires alteration of the sinogram. In the sinogram, each pixel represents the percent time a given leaf is open during a given projection (51 projections per gantry rotation, each projection approximately 7°). Thus, during every projection every leaf that opens also closes. By changing this value it causes the leaves to be open for too long or too short (e.g., because of changes in relay or fast/slow leaf movement). The authors would like to note that the study makes no implication into the possibility or frequency of such errors, and that they are simply experimental. However, as the integrity of the plan delivered depends on the synchronization of each independent component, by altering the timing of each, several possible failure modes are simulated. Each failure mode was tested independently and, when altering one component, the other two were left at the originally planned values.

For each patient plan, the ArcCHECK device was placed on the table and aligned to the laser positions of the unmodified DQA plan. The on‐board megavoltage CT (MVCT) was then used to fine‐tune the alignment. A typical adjustment was within 2 mm in any direction. All modified delivery plans based on that clinical plan were then delivered without moving the phantom, except to return the couch to its original position between each procedure. The tomotherapy unit was given several minutes of rest after each 15 min of delivery time.

**Figure 1:**
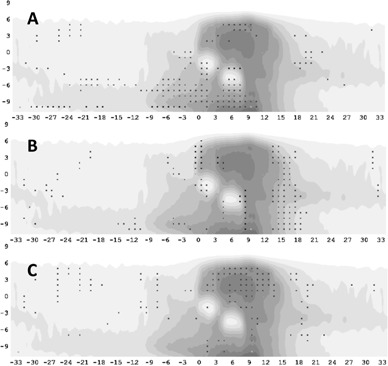
Gamma analysis failures due to alterations in (a) couch speed, (b) gantry synchronization, and (c) leaf open time. The severity of the alteration is such that the passing rate has fallen below 90% for 3%/3 mm gamma criteria based on the magnitude of the global maximum. The shading represents the expected dose (darker is a higher dose), while dots represent failing points. Each axis is centimeters along the length (vertical) and circumference (horizontal) of the ArcCHECK phantom.

Gamma analyses of the absolute dose maps were performed within the SNC patient software for both altered and unaltered plans (Fig. 1). When the ArcCHECK phantom is positioned such that the high‐dose region is on one side of the detector ring, a low dose is delivered to the opposite side of the ring, ranging from 15%–50% of the maximum dose, depending on the type of plan. In order to include these measurements, the threshold for analysis was set at 10% of the global maximum, excluding only detectors which were beyond the superior–inferior extent of the plan. The “3D distance to agreement,” “Tomotherapy measurement mode,” “Region of interest analysis,” and “Apply measurement uncertainty” options were used for all analyses. These options are more fully explained in the software manual, and respectively correct for the curved distance between diodes, disable angular, and field size corrections due to incompatibility with tomotherapy, and allow for 1% error in diode measurements alone. Results were tabulated for the use of both local magnitudes for gamma calculation and global (”Van Dyk”) percent of max dose.^(10)^

In addition to the plans presented into the study, the ArcCHECK gamma passing for 21 previously implemented plans of all types were tabulated. In accordance with the general practice in our clinic, the data collection for these analyses was performed by aligning phantom to the lasers, without MVCT alignment. To account for misalignment, autoshifts were performed within the software and 3%/3 mm gamma criteria were used.^(11)^ Autoshifting asks the software to find the best‐fit agreement between measured and expected plans by shifting the measured points 1 mm at a time, to correct for setup error. These 21 plans were clinically used and passed previous QA methods, and are intended to provide a reference of passing ranges for comparison.

The gamma passing rates for a 3%/3 mm criteria were tabulated for each of the nine original plans used in the study and the altered plans with manufactured errors. To determine the level of an altered parameter at which a plan would “fail” under clinical action levels, the size of error required to produce a gamma passing rate of under 90% for each site was calculated using linear interpolation of the acquired gamma rate data. The 90% passing rate is an action level at approximately the mean passing rate of previously treated plans in our clinic minus two standard deviations. In our clinical experience, this level is relatively insensitive to minor daily variations in output, but will confirm larger drifts in output and energy as determined by more direct machine QA.

## III. RESULTS

**Figure 2:**
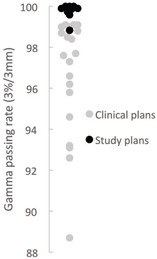
Spread of passing rates. The graph depicts the passing rates of the previously implemented clinical plans (without image‐guided alignment) across all plan types. The quartiles were 96%, 98%, and 99%. Also depicted are the unaltered plans from the current study which did employ image guidance to align the ArcCHECK phantom. This demonstrates the spread of passing rates across plans with a laser‐aligned ArcCHECK phantom versus image guidance.

All unmodified and modified procedures were deliverable on the machine and analyzed using ArcCHECK. Amongst the 21 previously analyzed plans, without imaging alignment of the phantom and autoshifted, 3%/3 mm gamma criteria, the minimum, lower quartile, median, and upper quartile passing rates were 89%, 96%, 98%, and 99%, respectively. The nine plans which were the subject of the present study, all had passing rates of at least 98% when unmodified (Fig. 2), using Van Dyk passing rates.

Plan alterations yielded deliverable procedures for the tomotherapy unit. When going through the plan alteration with a zero‐change (i.e., manual generation of intact plan), the passing rate was equivalent to those generated directly by the DQA process. It was found that increases and decreases of a given parameter (i.e., couch moving slower or faster) of the same magnitude yielded very similar passing rates, and were thus averaged for simplicity of analysis for the rest of the study. The data for each treatment site and each error type and magnitude were then averaged for presentation, noting the minimum and maximum values for each n=3 data point.

**Figure 3:**
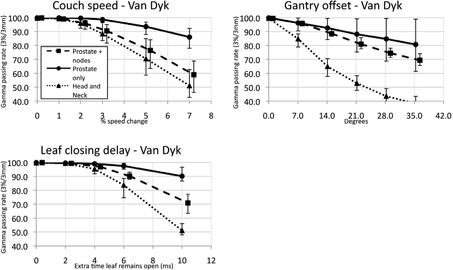
Degradation of passing rates with plan changes using global (Van Dyk) passing criteria. Each point represents the average gamma passing rate at 3%/3 mm for the type of plan (grouped by treatment site), for that magnitude of change. The error bars represent the maximum and minimum passing rates about that point.

**Figure 4:**
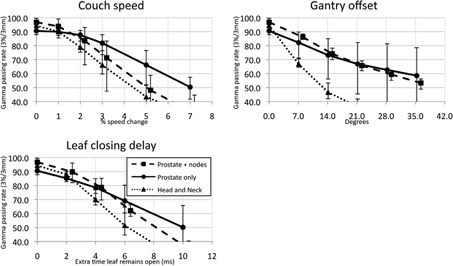
Degradation of passing rates with plan changes using the local magnitude of each dose measurement for gamma passing criteria. Each point represents the average gamma passing rate at 3%/3 mm for the type of plan, for that magnitude of change. The error bars show the maximum and minimum passing rates about that point. This test is more sensitive than global gamma rate, but with lower intact plan passing rates.

For every plan alteration made, there was a decrease in gamma passing rate correlated to the magnitude of the alteration. This falloff occurred at larger perturbations when analyzing data using global gamma passing criteria (Fig. 3) than with local calculations (Fig. 4). These changes were generally undetectable when very minor, and then hit a threshold at which the passing rate would drop quickly. The magnitude of each change required to degrade the passing rate to 90%, the action level in our clinic, is tabulated in Table 1 for gamma analysis using 3%/3 mm criteria with global magnitudes.

**Table 1:** Magnitude of change required to degrade passing rate to 90% or lower with global 3%/3 mm gamma analysis

	*Couch Speed (%)*	*Gantry/leaf Synch (°)*	*Leaf Closing Time (ms)*
Prostate+nodes	3.3	13.8	6.4
Prostate−only	5.9	18.2	10.0
Head&Neck	2.8	4.6	4.9

## IV. DISCUSSION

The ArcCHECK diode array allows for convenient acquisition of a nonplanar array of doses, and the cylindrical geometry is similar to the widely accepted cheese phantom. However, existing standards for gamma passing rates may need to be examined with the different sampling distribution as compared to the plane‐and‐point of the cheese phantom. In this study, we explored the impact of plan alterations on the gamma passing rate to elucidate the sensitivity of our clinic's DQA process, using the commonly employed 3%/3 mm passing criteria^(11)^ with an action level of 90% passing.

While the typical DQA analysis does not always include the imaging step for phantom alignment, it was employed in this study so as not to let positioning errors confound the data. Imaging‐based alignment is able to easily and natively detect necessary shifts in five axes, and with use of external BBs, it can be used for all six degrees of freedom (translations and roll to be performed within the tomotherapy system, and yaw and pitch rotations by hand). It was found that with this extra step, the gamma passing rates are very high, 98% and higher for the cases analyzed in the study (Fig. 3). In some circumstances, it is common practice in such analyses to “shift” the measured data with respect to the expected data, in order to minimize the impact of phantom‐positioning errors. The ArcCHECK analysis software can perform this shift in two axes: superior–inferior translation and roll. We found that with inclusion of the imaging step, autoshifting offers very little to improve results with the unaltered patient plans, yet caused some altered plans to improve from very low passing rates to the acceptable range (55% to 90% in the worst case, when a large error in couch speed was compensated for by an axial shift of dose distributions on a short plan). This suggests that autoshifting should not be conducted blindly as part of the analysis process. If employing imaging in the phantom setup, it should be unnecessary entirely. With a laser‐based setup, the shifts should be no larger than the tolerance of lasers in periodic QA.

The positioning of the phantom with respect to the planned high‐dose region will impact the appearance and sensitivity of the dose distribution measured. Neilson et al.^(12)^ provide an extensive comparison of the two in which they find that the passing rates of the two are similar, with the central positioning of the high‐dose region being less sensitive to analysis options (namely global versus local gamma magnitude calculations) and, therefore, recommended. In brief, the advantage of central positioning is that one may place an ion chamber in the center, and the diodes end up with comparable magnitudes of dose throughout the circumference barring regions of avoidance. However, the medium and lower level dose regions resulting from tomotherapy delivery are highly spatially variable (compared to volumetric‐modulated arc therapy deliveries using a standard linac) due to the frequent opening and closing of leaves throughout the rotation of the gantry. By offsetting the phantom and putting several diodes through a higher dose region, one achieves a smoother, more intuitive, dose distribution, including diode sampling at the approximate prescription dose. The drawbacks of positioning the phantom offset from the center include sacrificing the central ion chamber measurement, the more normal incident radiation, and introducing sensitivity to analysis parameters. With the offset phantom positioning, the threshold and global versus local gamma magnitude can impact gamma passing rates immensely. With global (a.k.a. Van Dyk) analysis, one may inadvertently apply tolerances of over 15% in the low‐dose regions, while local analysis will yield a higher error rate in the low‐dose regions and a lower overall passing rate. Ultimately, the choice of analysis parameters will impact a choice of action levels for passing rates, and inspection of the location and types of discrepancies will be more robust than automated analysis for individual measurements. However, gamma passing rates may remain useful for looking at trends. By using local magnitudes for gamma calculations, one has a more sensitive test but with generally lower passing rates for intact plans. This holds particularly true for the prostate‐only plans where the dose is highly localized, and the low doses on the opposite side of the phantom exhibit errors which are large compared to local measurements, but small compared to the global maximum.

When alterations are made to the plan, the dose measured compares unfavorably to the expected dose, increasing with the magnitude of the alterations. The errors manifest in an intuitive way. When the leaves remain open for too long, there is an increase in delivered dose which can be estimated by the overall increase in fluence. Plans with either a high gantry period or low total number of projections (leading to high leaf‐open times from each projection) are less sensitive to leaf‐open time changes. For reference, a typical leaf‐open time is approximately 200 ms, and it takes approximately 20 ms for a leaf to either open or close. While opening‐time errors of this type are likely to manifest in a far more random way (only one or two leaves are likely to travel slower at a time), a comprehensive delay evaluates a broader robustness to such changes. The more complex head and neck plans were consistently the most sensitive, requiring only a universal 5 ms addition to leaf opening time before the passing rate fell below 90%. Prostate‐only plans, on the other hand, have very few leaves open at a time that tend to remain open for most of a projection, and thus exhibit minor changes to passing rate at the same 5 ms level (Fig. 4).

When the couch speed is changed, it essentially “squeezes” or “stretches” a plan, having an impact on the overall dose, as well as the dose geometry, which worsens at the end of the plan as the superior–inferior displacement increases. Thus, it is as expected that the shorter prostate‐only plans demonstrate a lower sensitivity to such changes. There is also an incidental dependence on passing rate to the location of the higher dose area along the superior–inferior axis because of the increasing displacement as distance is covered.

As the gantry synchronization changes (thereby causing a rotational shift in the fluence around the S–I axis), it is the rotationally symmetric plans (e.g., prostate‐only) which exhibit the least sensitivity. However, this is only the case if the machine isocenter is in the middle of the target. It was observed in one case where the isocenter was at the border of the prostate, thereby demanding different leaf openings from different gantry angles, that the sensitivity was comparable to the more rotationally complex plans. On average, however, errors in head and neck plans were consistently detectable beyond a 5° offset, while prostate‐only plans could pass at very large perturbations greater than 15°.

The plans with the lowest sensitivity overall were the prostate‐only plans, in which the target structure was roughly axially symmetric. Conversely, the most sensitive plans were the head and neck plans, in which the fluence varies widely both with gantry angle and couch position (corresponding to anatomical superior–inferior). This makes intuitive sense, although it is unclear whether this is because the plans themselves are more robust (i.e., small delivery errors do not drastically change the dose distribution delivered) or because the DQA process used is not suited to detecting them. Further investigation using multiple detection styles may be able to stratify these components. A recent study by Fredh et al.^(13)^ exploring similarly manufactured errors on a Varian linear accelerator (Varian Medical Systems, Palo Alto, CA) delivering RapidArc plans found a wide variation between QA devices and their ability to detect plan changes. Generally, the sensitivity to errors will increase with physical length of plan, targets off‐center from the machine isocenter, and noncylindricality.

In depending on gamma passing rate to catch possible delivery errors, it appears that one error must be quite large before it causes rates to drop below a threshold of, for example, 90%. The leaves must be delayed or sped up by several milliseconds, the couch speed by several percent, and the gantry angle by several degrees (depending on the plan) before it is reflected in an ArcCHECK based gamma analysis. The tomotherapy treatment machine interlocks are designed to catch errors of a fraction of these magnitudes. Altering the analysis criteria by either using local magnitudes for gamma calculations or lowering the criteria to 2%/2 mm will make the test more sensitive. This will also lead to a lower passing rate on intact plans, requiring closer inspection of results. For example, a plan passing 85% of points may be acceptable if measurements not passing are in regions far from the target or critical structures, but undesirable otherwise, requiring interpretation by a qualified individual.

## V. CONCLUSIONS

In the normal course of planning and delivery QA, it is a combination of errors which contribute to less‐than‐perfect measurements, including dose calculation engines, phantom placements, and delivery errors. The findings presented in this study suggest that an ArcCHECK‐based gamma‐type DQA process may be more suitable for measuring this combination of errors than for gauging specific machine errors. It may also be possible to increase the sensitivity of the process by raising the threshold of gamma passing acceptability, taking extra care in setting up the phantom and not adjusting the expected data to best fit the measurements.

## ACKNOWLEDGMENTS

The authors would like to thank Mr. Mark Geurts at Accuray for his assistance in extracting the pertinent plan data from the file structure.
